# Long-term prognostic value of ^18^F-FLT PET/CT versus ^18^F-FDG PET/CT in locally advanced oesophageal squamous cell carcinoma: a prospective study

**DOI:** 10.1186/s40644-026-01056-2

**Published:** 2026-06-05

**Authors:** Wenyan Gao, Jingwen Peng, Xue Chen, Yi Wang, Shunji Zhi, Lei Wu, Gang Wan, Zhuzhong Cheng, Qifeng Wang, Wei Zhang

**Affiliations:** 1 Department of Radiation Oncology, Sichuan Clinical Research Center for Cancer, Sichuan Cancer Hospital & Institute, Sichuan Cancer Center, School of Medicine, University of Electronic Science and Technology of China, Chengdu, China; 2 Department of Radiation Oncology, Radiation Oncology Key Laboratory of Sichuan Province, Sichuan Clinical Research Center for Cancer, Sichuan Cancer Hospital & Institute, Sichuan Cancer Center, University of Electronic Science and Technology of China, Chengdu, China; 3 Cancer Center, Sichuan Taikang Hospital, Tianfu New Area, #881 Xiang He First Street, Chengdu, Sichuan Province China; 4 Department of Nuclear Medicine, Sichuan Clinical Research Center for Cancer, Sichuan Cancer Hospital & Institute, Sichuan Cancer Center, University of Electronic Science and Technology of China, Chengdu, China

**Keywords:** ^18^F-FLT, ^18^F-FDG, Oesophageal squamous cell carcinoma, Positron emission tomography, Prognosis

## Abstract

**Background:**

Accurate prognostication in locally advanced oesophageal squamous cell carcinoma (LA-ESCC) remains a significant clinical challenge. This prospective study aimed to compare the predictive value of fluorine-18 fluorodeoxyglucose (^18^F-FDG) and fluorine-18 fluorothymidine (^18^F-FLT) positron emission tomography/computed tomography (PET/CT) regarding long-term clinical outcomes.

**Methods:**

Between June 2018 and July 2019, 31 patients with LA-ESCC were prospectively enrolled and underwent both ^18^F-FDG and ^18^F-FLT PET/CT scans prior to or during initial therapy. Quantitative parameters, including maximum standardized uptake value (SUV _max_), minimum SUV (SUV _min_), mean SUV (SUV _mean_), tumour metabolic volume (MTV), total lesion glycolysis (TLG), and total lesion ^18^F-FLT expression (TLT), were measured for primary tumours and regional lymph nodes. Progression-free survival (PFS) and overall survival (OS) were analysed using Cox univariate regression, receiver operating characteristic (ROC) curve analysis, and Kaplan-Meier survival estimates.

**Results:**

In ^18^F-FLT PET/CT, primary tumour parameters (SUV _max_, SUV _mean_, TLT) and all lymph node parameters significantly distinguished between favourable and poor PFS groups (all P <0.05). Primary tumour SUV _max_, SUV _mean_, and all lymph node parameters demonstrated a negative correlation with both PFS and OS. Specifically, lymph node SUV max (PFS: hazard ratio (HR)=1.65, 95% confidence interval (CI):1.23–2.19, P =0.002; OS: HR = 1.81, 95% CI:1.29–2.55, P <0.001) and SUV mean showed strong predictive value. In contrast, for ^18^F-FDG PET/CT, only lymph node MTV differed significantly between PFS groups (P =0.03), with no parameters showing significant prognostic correlation. ROC analysis indicated that ^18^F-FLT lymph node SUV _max_ and SUV _mean_ yielded the highest predictive accuracy for PFS (area under the curve (AUC): 83.8% and 84.2%, respectively) and OS (AUC: 79.3% for both).

**Conclusions:**

^18^F-FLT PET/CT may offer superior prognostic value compared to ^18^F-FDG PET/CT for long-term survival prediction in LA-ESCC. Parameters derived from metastatic lymph nodes, particularly SUV _max_ and SUV _mean_, may serve as potential biomarkers for poor clinical outcomes, independent of imaging timing.

## Background

Oesophageal cancer (EC) ranks as a major digestive malignancy, characterized by high mortality and morbidity rates globally. Oesophageal squamous cell carcinoma (ESCC) represents the dominant histological subtype, accounting for nearly 90% of cases in Asia. Clinically, the disease poses a significant challenge as over two-thirds of patients present with metastatic or locally advanced disease at the time of diagnosis [1, 2]. Neoadjuvant chemoradiotherapy (NCRT) followed by surgery serves as the standard therapeutic regimen for locally advanced ESCC (LA-ESCC) [3]. For these patients, precise staging, timely detection of tumour recurrence and progression, and the subsequent adjustment of treatment strategies are of crucial importance [4, 5]. Molecular imaging utilizing positron emission tomography (PET) allows for the characterization of tumour properties based on biochemical and biological features, assessing underlying physiological processes [6–9]. In patients with EC, integrated fluorine-18 fluorodeoxyglucose (^18^F-FDG) PET/CT is well established to provide complementary diagnostic information to contrast-enhanced computed tomography (CT), particularly for lymph node staging and the detection of distant metastases [10, 11]. ^18^F-FDG PET/CT, utilizing the most common oncologic radiotracer, is also effective in detecting recurrent ESCC [12]. Furthermore, ^18^F-FDG uptake has shown correlations with neoadjuvant therapy response and overall survival in patients with EC [13–15], and several studies have indicated its utility in predicting prognosis [16–18]. Nevertheless, ^18^F-FDG PET/CT suffers from limitations in differentiating post-treatment peritumoral inflammation from residual malignant disease, as both tumour cells and inflammatory tissues exhibit high glucose metabolism and consequent FDG uptake [19].

In contrast, fluorine-18 fluorothymidine (^18^F-FLT), a thymidine analogue, is phosphorylated by thymidine kinase 1 (TK1) and trapped within cells specifically during the S phase of the cell cycle. As a surrogate marker of DNA synthesis, ^18^F-FLT possesses greater specificity for malignancy and is significantly less influenced by inflammatory processes than ^18^F-FDG. Empirical studies suggest that ^18^F-FLT is more effective in evaluating therapeutic responses in lymphoma, breast cancer, and ESCC [20–24]. A previous pilot study indicated that it might offer superior predictive value for therapeutic response in EC [25].

However, literature regarding the relationship between ^18^F-FLT PET/CT parameters and *long-term* prognosis in patients with LA-ESCC remains scarce. The present study aimed to ascertain and compare the predictive value of ^18^F-FLT PET/CT and ^18^F-FDG PET/CT parameters regarding long-term prognosis in patients with LA-ESCC. The ultimate goal was to identify high-risk patients through quantitative imaging to facilitate individualized tumour treatment strategies.

## Materials and methods

### Patient cohort

**Fig. 1:**
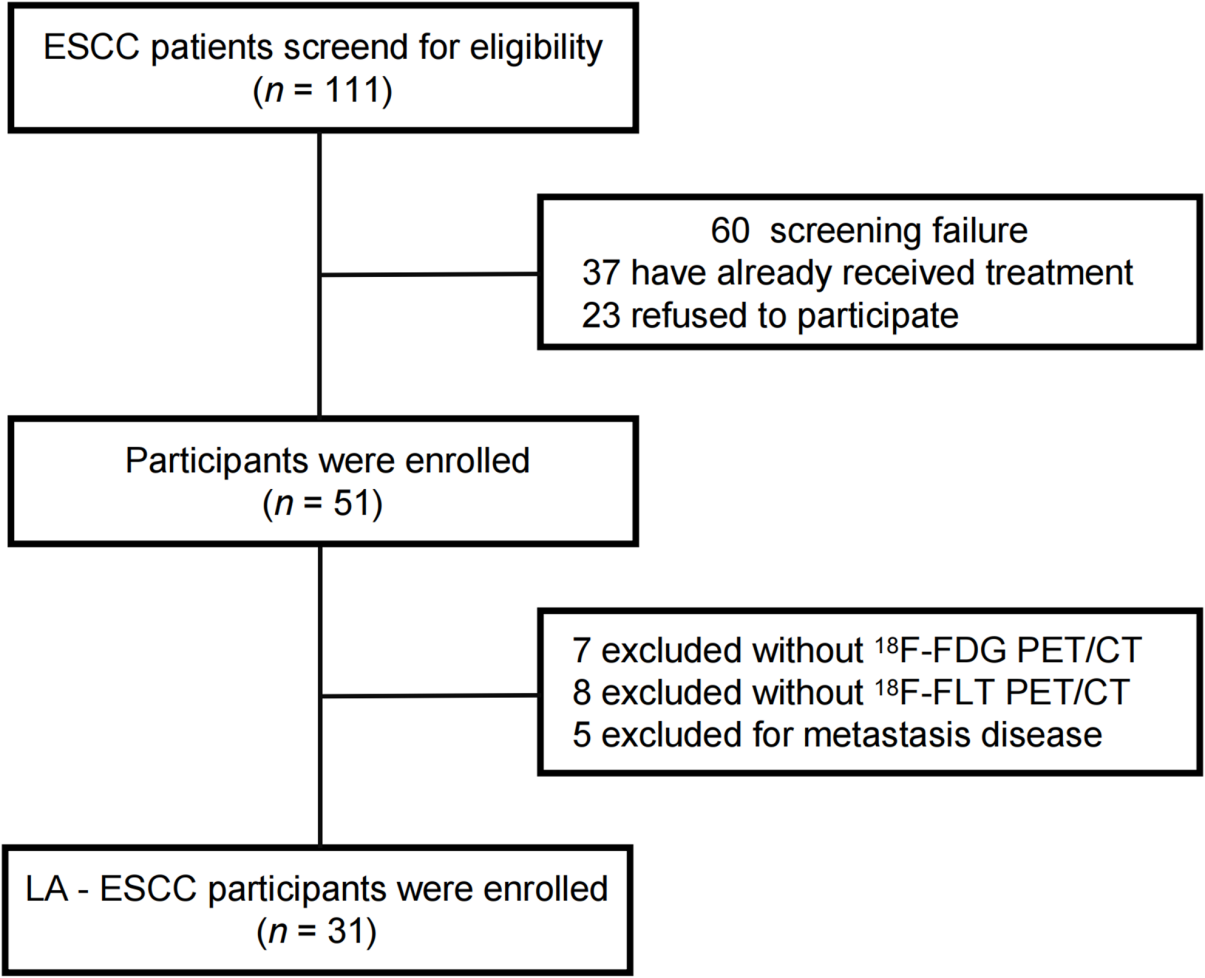
Research flowchart. Diagram illustrating patient recruitment, inclusion/exclusion criteria, and study procedures

This prospective cohort study recruited eligible patients with LA-ESCC from the Sichuan Cancer Hospital and Research Institute between June 2018 and July 2019. The study protocol was approved by the Ethics Committee of the Sichuan Provincial Cancer Hospital and Research Institute (Study registration number: 2018SZ0210; Ethical review batch number: SCCHEC-02-2019-003). This work was supported by the Science and Technology Department of Sichuan Province (Grant Nos. 2023YFS0488 and 2023YFQ0055). All patients underwent comprehensive pretreatment evaluations and provided written informed consent. The study design flowchart is presented in Fig. 1.

## Inclusion and exclusion criteria

Inclusion criteria were: (1) histologically confirmed oesophageal squamous cell carcinoma; (2) TNM stage T2–4 N1–3 M0; (3) normal pre-treatment haematological and biochemical indices; (4) age between 18 and 75 years; and (5) provision of signed informed consent. Exclusion criteria were: (1) prior chemotherapy or radiotherapy; (2) unwillingness to participate or inability to cooperate; (3) uncontrolled infection or severe medical conditions; and (4) inability to tolerate therapy.

## Treatments

All patients received either NCRT followed by surgery or radical concurrent chemoradiotherapy (CCRT), depending on surgical eligibility. The total radiotherapy dose ranged from 40 to 66 Gy, delivered using intensity-modulated radiation therapy (IMRT) fractionated at 1.8–2.0 Gy per day, 5 days per week. The chemotherapy regimen was primarily based on paclitaxel combined with platinum agents (carboplatin, cisplatin, or nedaplatin). Specific chemotherapy regimens are detailed in Table 1. Oesophagectomy was performed 5–9 weeks post-NCRT. All postoperative specimens were evaluated for tumour sections, resection margins, depth of invasion, and regional lymph nodes. Tumour grade and stage were determined according to the 8th edition of the American Joint Committee on Cancer (AJCC) TNM staging system [26].

**Table 1:**
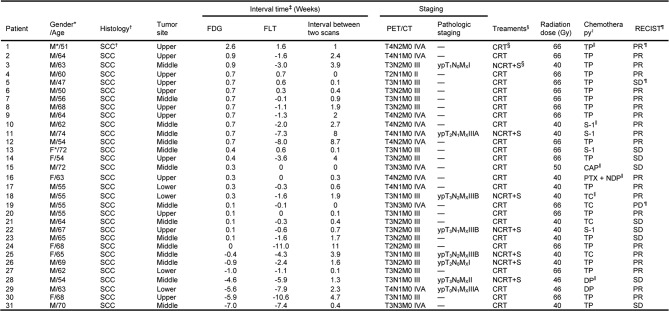
Patients’ characteristic

Abbreviations: *M = Male, *F = Female; SCC† = Squamous cell carcinoma; Interval time‡ = Time from ^18^F – FDG /^18^F - FLT PET/CT scans to treatment initiation.CRT§ = Chemoradiotherapy, NCRT + S§ = Neoadjuvant Chemoradiotherapy + Surgery; TP ǁ = Paclitaxel + cisplatin; S – 1ǁ = Tegafur, Gimeracil and Oteracil Potassium; CAPǁ = Capecitabine; PTX+NDPǁ = Paclitaxel + nedaplatin; TCǁ = Paclitaxel + carboplatin; DPǁ = Docetaxel + cisplatin; ǁ, RECIST criteria: PR¶= partial response, SD¶ = stable disease, PD¶ = progressive disease

## ^18^F-FDG PET and ^18^F-FLT PET scans

### ^18^F-FDG PET and ^18^F-FLT PET acquisition

Thirty-one patients diagnosed with LA-ESCC underwent both whole-body ^18^F-FDG PET and ^18^F-FLT PET scans during the initial treatment phase. Scans were reviewed by two experienced nuclear medicine physicians. Both tracers (^18^F-FDG and ^18^F-FLT) were prepared at the Sichuan Cancer Hospital PET/CT Centre. ^18^F-FDG and ^18^F-FLT were synthesized according to the method described by Wang et al., with radiochemical purity maintained at > 95% [27]. Imaging was performed using a Siemens Biograph mCT 64 PET/CT scanner (Siemens Healthineers, Erlangen, Germany). For ^18^F-FDG imaging, patients fasted for at least 6 h, ensuring blood glucose levels were < 11.1mmol/L. An intravenous injection of ^18^F-FDG (3.7–5.5 MBq/kg) was administered, followed by a 60-minute uptake period. For ^18^F-FLT imaging, patients received an intravenous injection of 340–450 MBq (total dose) without a fasting requirement, and PET/CT imaging was performed 60 min post-injection.

Baseline scans were conducted in whole-body mode, and follow-up scans were partial-body (seventh cervical vertebra to abdomen). CT scan acquisition parameters were 120 kV, 200 mA, and matrix 512 × 512, with a 1.5-minute acquisition time per bed position and an axial slice thickness of 5 mm. Images were reconstructed using the ordered subset expectation maximization (OSEM) algorithm following CT-based attenuation correction.

Two trained nuclear medicine physicians, blinded to clinical outcomes, performed visual and semi‑quantitative analyses using a dedicated nuclear medicine workstation (Hermes Medical Solutions, Stockholm, Sweden). For the primary tumour and positive lymph nodes, region of interest (ROI) delineation was performed slice‑by‑slice along the tumour boundary on the fused PET/CT volume to accurately define the gross tumour volume (GTV). A threshold of 40% of the maximum standardized uptake value (SUV _max_) of the entire lesion was applied as an isodose contour line for precise tumour delineation. Metastatic lymph nodes were also included. The following quantitative parameters were then automatically calculated: maximum standardized uptake value (SUV _max_), mean SUV (SUV _mean_), minimum SUV (SUV _min_), metabolic tumour volume (MTV), total lesion glycolysis (TLG), and total lesion ¹⁸F‑FLT expression (TLT).

## Clinical data collection

Follow-up was conducted every 3 months for the first 2 years, and every 6 months thereafter until death or study closure. Assessments included laboratory tests, contrast-enhanced chest CT, and PET/CT scans (when clinically indicated). The primary endpoints were overall survival (OS) and progression-free survival (PFS).

## Statistical analysis

Quantitative PET parameters (SUV _max_, SUV _mean_, SUV _min_, MTV, TLG, TLT) were expressed as mean ± SD. The Mann-Whitney U test was employed to compare PET/CT parameters between good PFS (PFS ≥ median) and poor PFS (PFS < median) groups divided by the median PFS of the entire cohort. Cox univariate regression analysis was used to assess the correlation between ^18^F-FDG and ^18^F-FLT PET/CT parameters, clinical variables, and long-term prognosis. Receiver operating characteristic (ROC) analysis was utilized to identify optimal cut-off values for PET parameters in predicting survival outcomes. Kaplan-Meier analysis and Cox proportional hazards models were used to compare 2-year PFS and OS rates. All statistical tests were two-sided, and a P-value < 0.05 was considered statistically significant. Statistical analyses were performed using SPSS software (version 25.0, IBM Corp., Armonk, NY, USA).

## Results

### Patient characteristics and outcomes

From a total of 111 screened individuals, 31 patients with LA-ESCC were enrolled between June 2018 and July 2019 (Fig. 1). The baseline demographic and clinical characteristics of these patients are summarized in Table 1, including age, sex, tumour location, and treatment details. Notably, Table 1 also presents PET/CT staging (based on combined interpretation of both ¹⁸F-FDG and ¹⁸F-FLT PET/CT scans), the time interval from each scan to treatment initiation, the interval between the two scans, pathological findings (ypT-NAM grade where available), and RECIST response classification. The median time from ^18^F-FDG PET/CT to the initiation of treatment was 0.3 weeks (range: -7 to 2.6 weeks), and − 1.3 weeks for ^18^F-FLT PET/CT (range: -11 to 1.6 weeks). A negative value indicates that the scan was performed after the treatment initiation. The median time interval between the ¹⁸F-FDG and ^18^F-FLT PET/CT scans was 1.3 weeks (range: 0–11.0 weeks; Table 1). The median follow-up duration was 62 months (range: 3–66 months). Median OS was 26 months (range: 3–66 months), and median PFS was 10 months (range: 2–66 months). At the censorship date, 22 patients (71.0%) had died, and 9 (29.0%) remained alive.

## ^18^F-FDG and ^18^F-FLT PET/CT parameter compariso

### Comparison of ^18^F-FDG and ^18^F-FLT PET/CT parameters

**Fig. 2:**
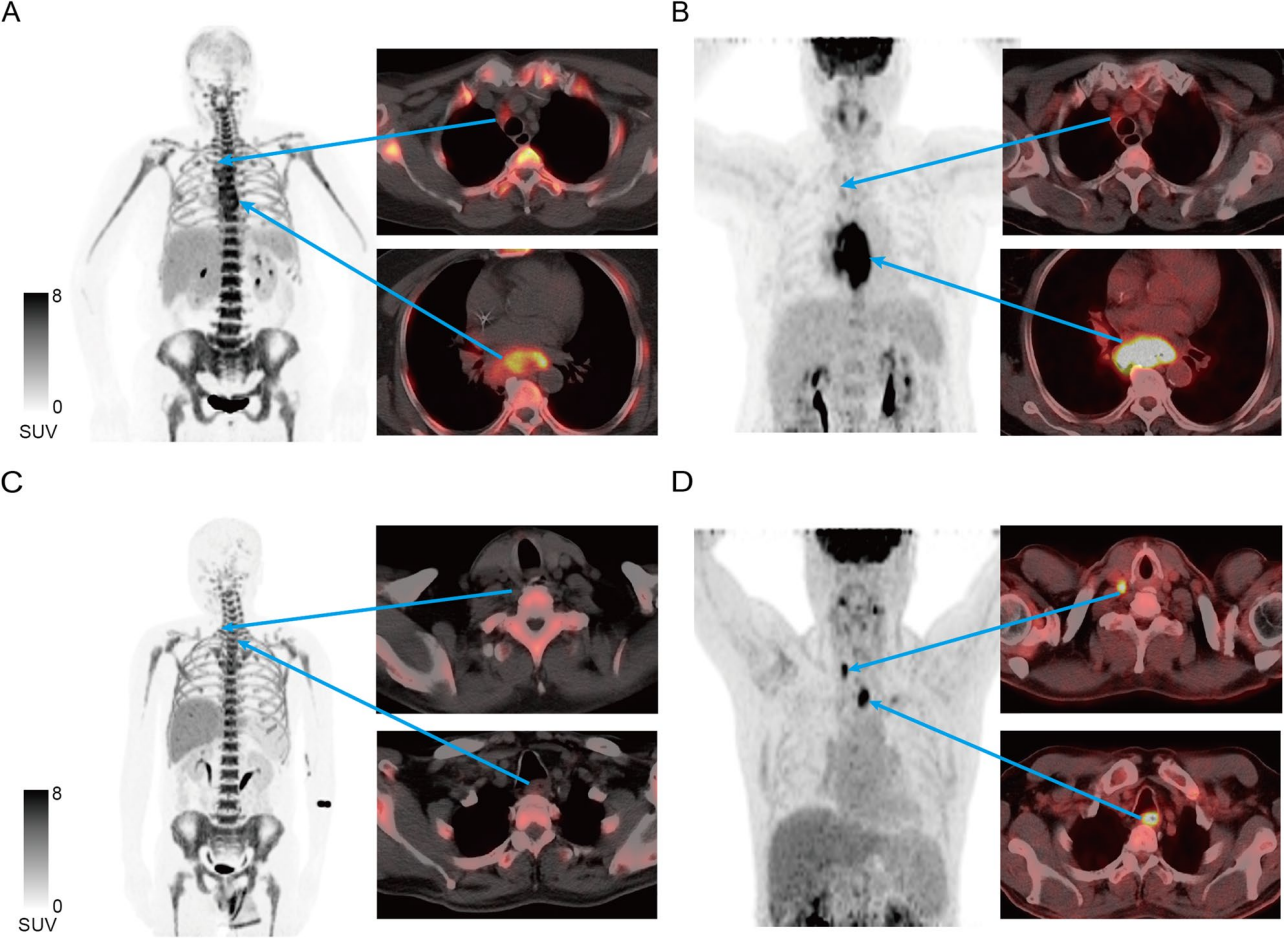
Representative ^18^F-FDG and ^18^F-FLT PET/CT images of two patients with locally advanced oesophageal squamous cell carcinoma (LA-ESCC). **A, B**: Patient 16 (poor prognosis): **A**: ^18^F-FLT PET/CT showing high uptake in metastatic lymph node and primary tumour; **B**: ^18^F-FDG PET/CT of the same patient showing high uptake in both lesions.**C, D**: Patient 4 (good prognosis): **C**: ^18^F-FLT PET/CT showing low uptake in both lesions; **D**: ^18^F-FDG PET/CT of the same patient showing high uptake in both lesions

Baseline PET/CT parameters stratified by PFS group according to the median PFS of 10 months (PFS ≥ 10 months = good; PFS < 10 months = poor) are presented in Table 2. Representative ^18^F-FDG and ^18^F-FLT PET/CT images from patients with poor and good prognoses are shown in Fig. 2. As anticipated, most ^18^F-FDG parameters (primary tumour SUV _max_, SUV _mean_, TLG, SUV _min_; lymph node SUV _max_, SUV _mean_, TLG) were lower in the ‘good PFS’ group (PFS ≥ 10 months) (all *P* > 0.05). In contrast, lymph node MTV, primary tumour MTV, and lymph node SUV _min_ were higher in the good PFS group; only lymph node MTV was significant (*P* = 0.03), while the other two were not (*P* = 0.423 and *P* = 0.101). Conversely, ^18^F-FLT parameters were significantly higher in the ‘poor PFS’ group (PFS < 10 months). Specifically, for ^18^F-FLT PET/CT, the SUV _max_, SUV _mean_, and TLT of the primary tumour, as well as all lymph node parameters, differed significantly between the prognostic groups (all *P* < 0.05).

**Table 2:** ^18^F-FDG and ^18^F-FLT PET/CT Parameter Comparison

Variables*	Total (*n* = 31)	Prognostic grouping^†^			
	Mean ± SD	Good PFS^†^ (*n* = 16)		Poor PFS^†^ (*n* = 15)	*P*
T^*^ FDG SUV _max_*	26.86 ± 52.20	17.83 ± 8.99		36.48 ± 74.57	0.984
T FDG SUV _min_*	2.70 ± 8.05	1.56 ± 1.73		3.91 ± 11.52	0.682
T FDG SUV _mean_*	17.91 ± 36.50	11.23 ± 5.22		25.03 ± 52.16	0.861
T FDG MTV*	9.70 ± 10.06	10.04 ± 8.15		9.35 ± 12.07	0.423
T FDG TLG*	149.02 ± 247.18	114.33 ± 101.49		186.03 ± 342.13	0.77
T FLT SUV _max_^‡^	7.31 ± 4.61	5.08 ± 3.89		9.40 ± 4.33	**0.006**
T FLT SUV _min_	0.69 ± 0.51	0.58 ± 0.45		0.80 ± 0.56	0.299
T FLT SUV _mean_^‡^	4.54 ± 2.75	3.03 ± 2.13		5.95 ± 2.54	**0.002**
T FLT MTV	6.97 ± 6.21	6.23 ± 7.27		7.67 ± 5.17	0.11
T FLT TLT* ^‡^	37.23 ± 51.11	28.6 ± 58.3		45.32 ± 43.68	**0.012**
LN^*^ FDG SUV _max_	27.66 ± 43.70	24.57 ± 14.94		30.96 ± 61.89	0.072
LN FDG SUV _min_	4.59 ± 5.08	4.62 ± 2.74		4.55 ± 6.87	0.101
LN FDG SUV _mean_	17.82 ± 27.18	16.12 ± 9.60		19.64 ± 38.44	0.072
LN FDG MTV^‡^	6.80 ± 7.59	7.91 ± 6.28		5.61 ± 8.86	**0.03**
LN FDG TLG	55.91 ± 101.92	37.68 ± 39.31		75.35 ± 140.78	0.202
LN FLT SUV _max_^‡^	10.46 ± 8.68	6.83 ± 8.06		13.86 ± 8.02	**0.004**
LN FLT SUV _min_^‡^	2.07 ± 1.55	1.43 ± 1.06		2.67 ± 1.72	**0.009**
LN FLT SUV _mean_^‡^	6.94 ± 5.71	4.5 ± 5.27		9.22 ± 5.28	**0.004**
LN FLT MTV^‡^	5.54 ± 5.97	3.35 ± 3.54		7.6 ± 7.09	**0.017**
LN FLT TLT* ^‡^	12.06 ± 14.73	5.90 ± 7.23		17.84 ± 17.65	**0.007**

Abbreviations: T* = Primary tumours; SUV _max_* = Maximum standard uptake values; SUV _mean_* = Mean standard uptake values; SUV _min_* = Minimum standard uptake values; MTV* = tumour metabolic volume; TLG* = Total lesion glycolysis (^18^F-FDG); TLT* = Total lesion FLT expression (^18^F-FLT); LN* = Positive lymph nodes; Good PFS† = Patients in PFS ≥ 10 months group, Poor PFS† = Patients in PFS<10 months group; ‡ *P* < 0.05

### Cox univariate regression analysis for PFS and OS

Table 3 presents the Cox univariate regression analysis of clinical variables. A liquid diet was associated with poorer PFS (hazard ratio (HR) = 3.11, 95% confidence interval (CI): 1.25–7.73, *P* = 0.016); and OS (HR = 2.75, 95% CI: 1.17–6.43, *P* = 0.022). Furthermore, N3 stage was identified as an adverse prognostic factor for both PFS (HR = 4.64; 95% CI: 1.09–19.69; *P* = 0.033) and OS (HR = 4.85; 95% CI: 1.18–19.95; *P* = 0.021). Tables 4 and 5 detail the analysis of PET/CT parameters. For the primary tumour on ^18^F-FLT PET/CT, SUV mean was a significant adverse prognostic predictor for both PFS (HR = 1.40; 95% CI: 1.16–1.68; *P* < 0.001) and OS (HR = 1.19; 95% CI: 1.03–1.39; *P* = 0.023). SUV max of the primary tumour also predicted poorer PFS (HR = 1.16; 95% CI: 1.06–1.27;* P* = 0.003). Notably, all ^18^F-FLT PET/CT lymph node parameters were adverse prognostic predictors for both PFS and OS. Specifically, lymph node SUV max showed a strong association with outcome (PFS: HR = 1.65, 95% CI: 1.23–2.19, *P* = 0.002; OS: HR = 1.81, 95% CI: 1.29–2.55, *P* < 0.001). In contrast, no ^18^F-FDG PET/CT parameters showed significant correlations with survival outcomes.

**Table 3:** COX Univariate Regression Analysis of Clinical Information for PFS and OS

Variables*	PFS			OS	
	HR (95% CI)	*P*		HR (95% CI)	*P*
Gender		0.111			0.442
Female	1			1	
Male	2.85 (0.65,12.45)			1.51 (0.51, 4.48)	
Age		0.839			0.568
<65	1			1	
≥ 65	0.91 (0.36, 2.31)			1.29 (0.55, 3.03)	
ECOG		0.619			0.348
1	1			1	
0	0.78 (0.29, 2.06)			0.64 (0.26, 1.58)	
Smoke		0.321			0.564
No	1			1	
Yes	1.65 (0.59, 4.59)			1.3 (0.53, 3.2)	
Drink		0.719			0.81
No	1			1	
Yes	1.19 (0.47, 3.01)			1.11 (0.47, 2.61)	
Diet^†^		**0.016**			**0.022**
Normal	1			1	
Liquid	3.11 (1.25, 7.73)			2.75 (1.17, 6.43)	
tumour site		0.497			0.439
Upper	1			1	
Middle	0.58 (0.22, 1.5)			0.6 (0.25, 1.46)	
Lower	0.58 (0.12, 2.68)			0.48 (0.11, 2.2)	
T stage		0.122			0.489
2	1			1	
3	73205578.11 (0, Inf)			2.31 (0.3, 17.68)	
4	96748764.66 (0, Inf)			3.06 (0.37, 24.99)	
N stage*^†^		**0.033**			**0.021**
1	1			1	
2	3.3 (1.14, 9.58)			3.15 (1.21, 8.18)	
3	4.64 (1.09, 19.69)			4.85 (1.18, 19.95)	
Stage		0.152			0.119
II	1			1	
III	59,339,818 (0, Inf)			62114178.64 (0, Inf)	
IVA	112537400.35 (0, Inf)			109496744.44 (0, Inf)	
Treatments		0.095			0.108
NCRT	1			1	
CRT	2.59 (0.75, 8.92)			2.28 (0.77, 6.75)	

Abbreviations: N stage* are diagnosed through ^18^F-FDG PET/CT and ^18^F-FLT PET/CT. † *P* < 0.05

**Table 4:** COX Univariate Regression of Primary tumour ^18^F-FDG and ^18^F-FLT PET/CT Parameters for PFS and OS

Variables	PFS			OS	
	HR (95% CI)	*P*		HR (95% CI)	*P*
T FDG SUV _max_	0.99 (0.97, 1.01)	0.191		0.98 (0.95, 1.02)	0.075
T FDG SUV _min_	0.96 (0.87, 1.07)	0.337		0.95 (0.85, 1.06)	0.192
T FDG SUV _mean_	0.98 (0.94, 1.03)	0.159		0.97 (0.91, 1.03)	0.061
T FDG MTV	1.001 (0.9596, 1.0441)	0.965		0.9939 (0.9528, 1.0367)	0.77
T FDG TLG	0.9987 (0.996, 1.0015)	0.278		0.9979 (0.9946, 1.0013)	0.107
T FLT SUV _max_^†^	1.16 (1.06, 1.27)	**0.003**		1.08 (1, 1.18)	0.069
T FLT SUV _min_	1.6 (0.7, 3.68)	0.287		1.06 (0.47, 2.38)	0.886
T FLT SUV _mean_^†^	1.4 (1.16, 1.68)	**< 0.001**		1.19 (1.03, 1.39)	**0.023**
T FLT MTV	1.02 (0.96, 1.08)	0.53		1.01 (0.95, 1.07)	0.747
T FLT TLT*	1.0035 (0.9967, 1.0102)	0.354		1.002 (0.995, 1.009)	0.596

Abbreviations: TLT* = Total lesion FLT expression (^18^F-FLT). † *P* < 0.05

**Table 5:** COX Univariate Regression of Positive Lymph Nodes ^18^F-FDG and ^18^F -FLT PET/CT Parameters for PFS and OS

Variables*	PFS			OS	
	HR (95% CI)	*P*		HR (95% CI)	*P*
LN FDG SUV _max_	0.997 (0.9856, 1.0085)	0.574		0.9966 (0.9863, 1.007)	0.471
LN FDG SUV _min_	1.0019 (0.9286, 1.0811)	0.96		0.9952 (0.9269, 1.0687)	0.894
LN FDG SUV _mean_	0.9958 (0.9781, 1.0137)	0.612		0.9949 (0.9787, 1.0113)	0.497
LN FDG MTV	1.02 (0.97, 1.07)	0.514		1.03 (0.99, 1.07)	0.235
LN FDG TLG	0.9968 (0.9911, 1.0026)	0.203		0.9978 (0.9933, 1.0023)	0.286
LN FLT SUV _max_^†^	1.07 (1.03, 1.12)	**0.002**		1.08 (1.03, 1.13)	**0.002**
LN FLT SUV _min_^†^	1.65 (1.23, 2.19)	**0.002**		1.81 (1.29, 2.55)	**< 0.001**
LN FLT SUV _mean_^†^	1.12 (1.05, 1.19)	**0.002**		1.13 (1.06, 1.21)	**0.001**
LN FLT MTV^†^	1.08 (1.01, 1.16)	**0.041**		1.08 (1.02, 1.15)	**0.024**
LN FLT TLT* ^†^	1.05 (1.02, 1.09)	**0.003**		1.04 (1.01, 1.07)	**0.012**

Abbreviations: TLT* = Total lesion FLT expression (^18^F-FLT). † *P* < 0.05

### Predictive accuracy of ^18^F-FLT PET/CT

**Fig. 3:**
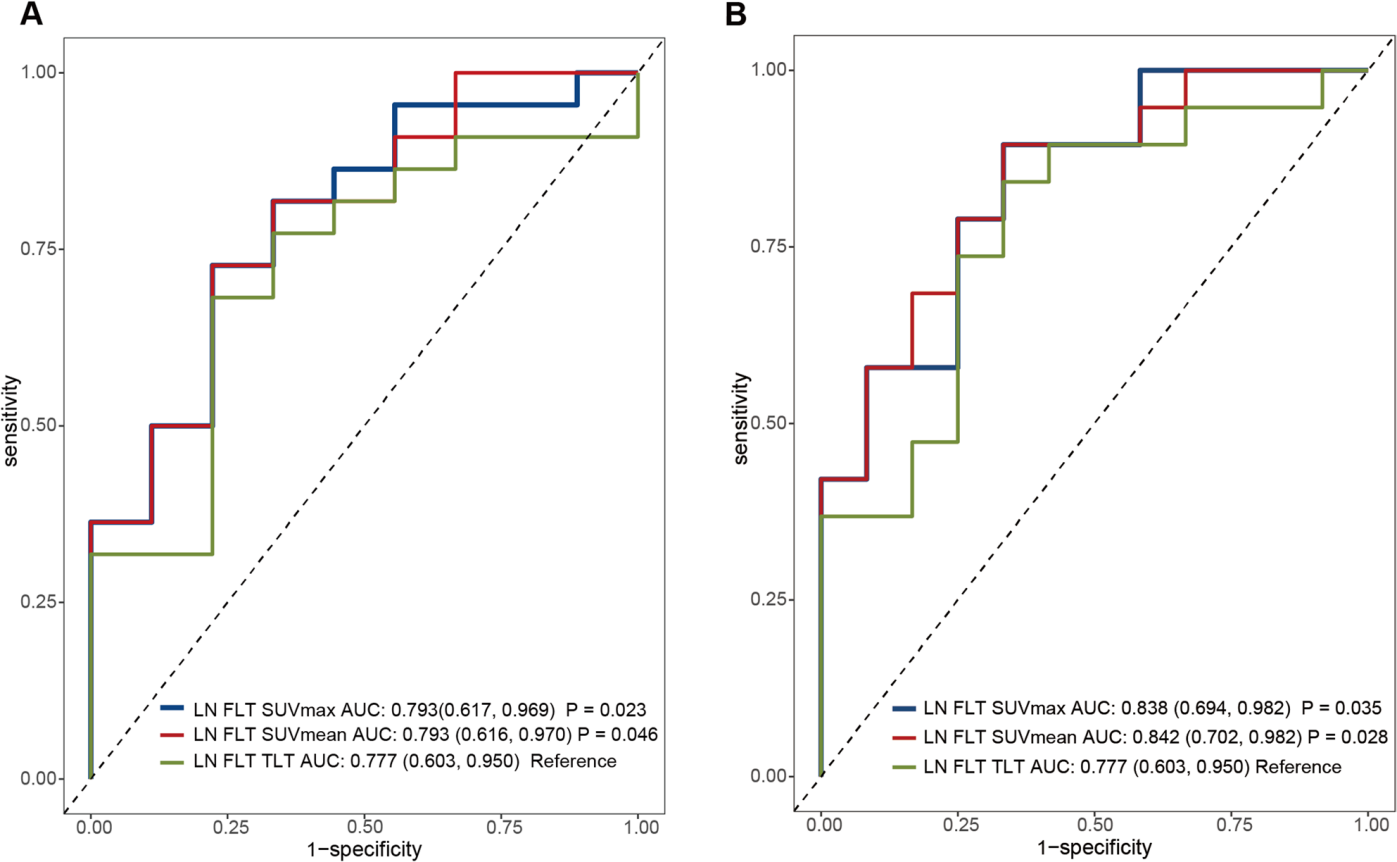
Receiver operating characteristic (ROC) curves of optimal ^18^F-FLT PET/CT lymph node parameters. **A**: SUV max and SUV mean for overall survival (OS). **B**: SUV max and SUV mean for progression-free survival (PFS)

**Fig. 4:**
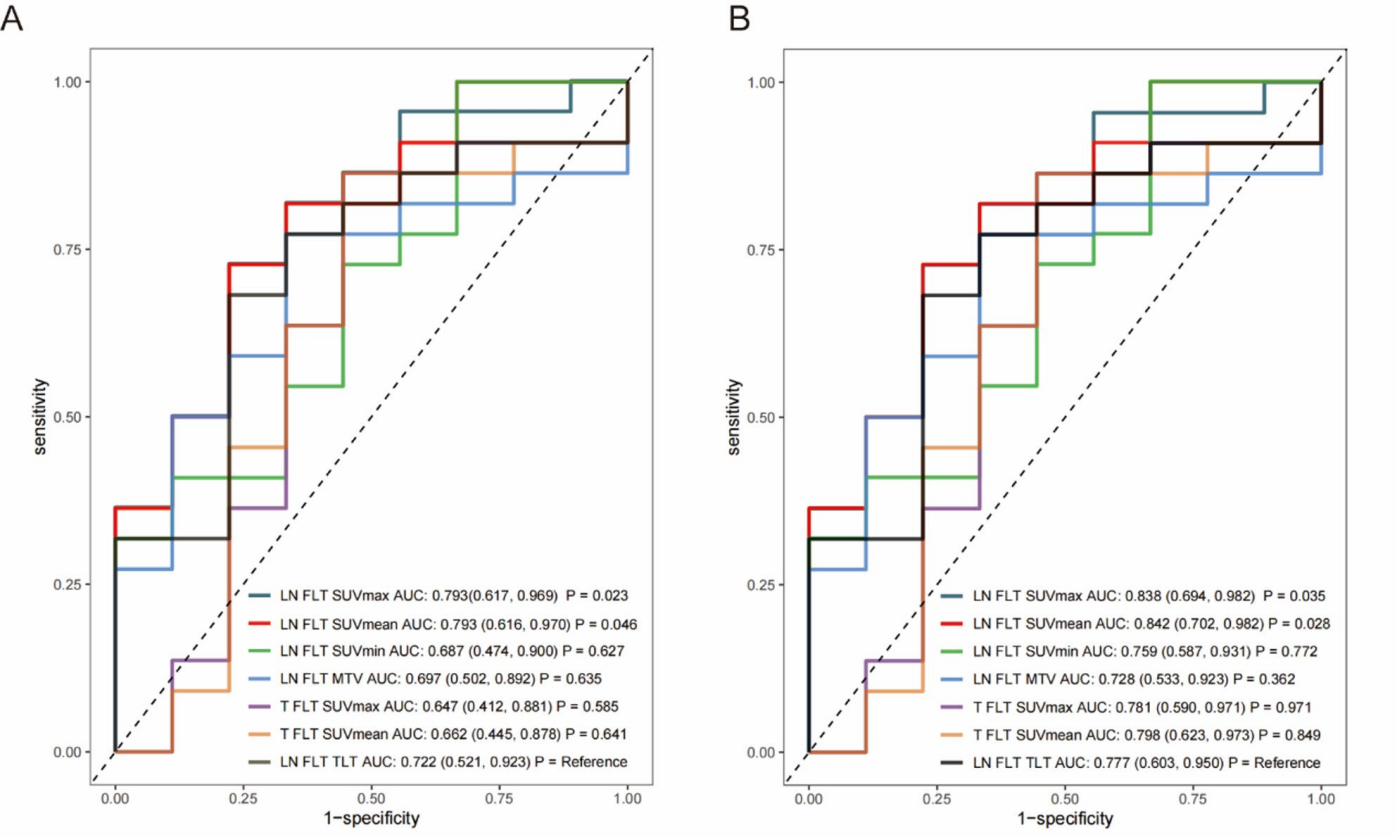
ROC curves of all prognostic-related ^18^F-FLT PET/CT parameters for predicting OS and PFS. **A**: ROC curves for OS prediction. **B**: ROC curves for PFS prediction. Parameters included lymph node (LN) FLT SUV max, SUV mean, SUV min, MTV, TLT, and primary tumour (T) FLT SUV max, SUV mean. Among these, only LN FLT SUV max and SUV mean reached statistical significance (see Figure 3)

**Fig. 5:**
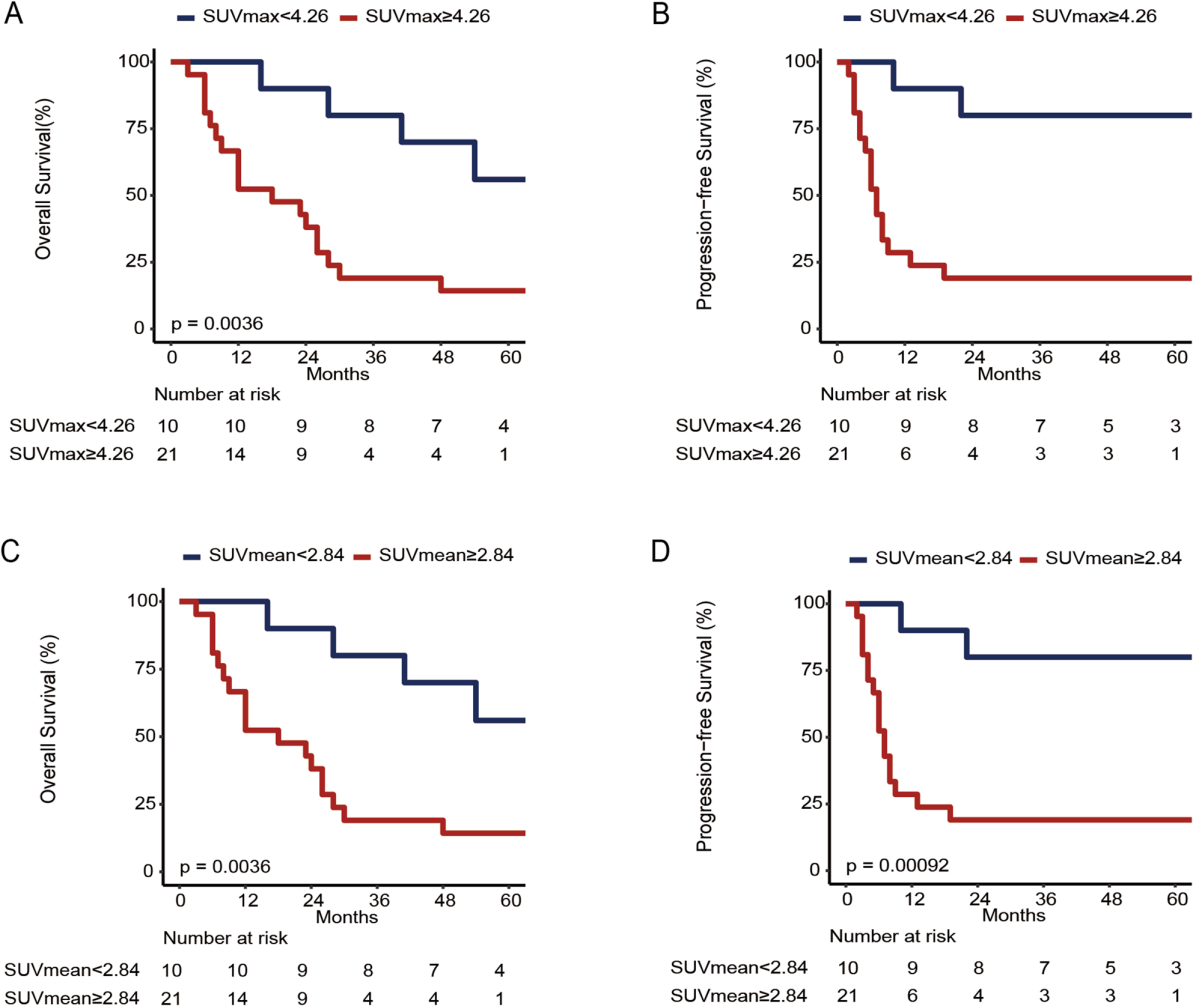
Kaplan–Meier survival curves stratified by optimal ^18^F-FLT PET/CT lymph node parameters. **A**: OS based on SUV max cutoff (4.26). **B**: PFS based on SUV max cutoff (4.26). **C**: OS based on SUV mean cutoff (2.84). **D**: PFS based on SUV mean cutoff (2.84)

The ROC curve analyses assessing the predictive accuracy of ^18^F-FLT PET/CT parameters are summarized in Table 6. The areas under the curve (AUCs) for lymph node parameters were consistently higher than those for primary tumour parameters for both PFS and OS. For example, lymph node SUV _max_ achieved an AUC of 83.8% for PFS and 79.3% for OS, compared to 78.7% and 64.7% for the primary tumour SUV _max_, respectively. ROC curve analysis revealed that lymph node ^18^F-FLT SUV _max_ and SUV _mean_ were significant predictors for both OS and PFS (Fig. 3; OS: AUC = 0.793, *p* = 0.023 and *p* = 0.046; PFS: AUC = 0.838 and 0.842, *P* = 0.035 and *P* = 0.028). The ROC curves for all the prognostic-related parameters are presented in Fig. 4 (Fig. 4A for OS, Fig. 4B for PFS).The optimal cut-off values determined by Youden indices were: primary tumour SUV max 3.66, SUV mean 2.27; and lymph node SUV max 4.26, SUV mean 2.84. Fig. 5 and Table 6 consistently demonstrate that patients with parameters below cut-offs (primary tumour SUV max 3.66, SUV mean 2.27; lymph node SUV max 4.26, SUV mean 2.84, SUV min 2.26, MTV 2.21, TLT 2.7) had better 2-year PFS and OS (all *P* < 0.05).

**Table 6:** Comparison of Prognostic Predictive Performance: ^18^F-FLT PET/CT Parameters

Variables*	Optimal cut-off value	*P*			AUC	
		2-year PFS (%)	2-year OS (%)		PFS (%)	OS (%)
T FLT SUV _max_^†^	3.66	71.4 vs. 29.2 ***P*** **= 0.036**	71.4 vs. 45.8 ***P*** **= 0.048**		78.7	62.1
T FLT SUV _mean_^†^	2.27	88.9 vs. 18.2 ***P*** **= 0.0013**	77.8 vs. 40.9 ***P*** **= 0.012**		79.8	62.1
LN FLT SUV _max_^†^	4.26	80 vs. 19 ***P*** **= 0.00092**	80 vs. 38.1 ***P*** **= 0.0036**		83.8	79.3
LN FLT SUV _min_^†^	2.26	52.4 vs. 10 ***P*** **= 0.0022**	66.7 vs. 30 ***P*** **= 0.0061**		75.9	68.7
LN FLT SUV _mean_^†^	2.84	80 vs. 19 ***P*** **= 0.00092**	80 vs. 38.1 ***P*** **= 0.0036**		84.2	79.3
LN FLT MTV^†^	2.21	72.7 vs. 35 ***P*** **= 0.0041**	72.7 vs. 20 ***P*** **= 0.003**		72.8	69.7
LN FLT TLT* ^†^	2.7	53.3 vs. 25 ***P*** **= 0.032**	73.3 vs. 37.5 ***P*** **= 0.048**		77.7	72.2

Abbreviations: TLT* = Total lesion FLT expression (^18^F-FLT). † *P* < 0.05

## Discussion

This prospective study evaluated the long-term prognostic utility of ^18^F-FDG and ^18^F-FLT PET/CT parameters in 31 patients with LA-ESCC. To our knowledge, this study features the longest follow-up period to date (median 62 months) among research addressing this specific comparison. The principal finding is that ^18^F-FLT PET/CT parameters, particularly those derived from metastatic lymph nodes, were significantly correlated with both PFS and OS, whereas ^18^F-FDG parameters failed to show similar prognostic power. Consequently, ^18^F-FLT PET/CT appears to offer superior value over ^18^F-FDG PET/CT in predicting long-term outcomes and stratifying high-risk patients.

As anticipated, quantitative uptake parameters were higher in ^18^F-FDG PET/CT than in ^18^F-FLT PET/CT for both primary tumours and lymph nodes [28]. This discrepancy stems from their distinct uptake mechanisms. ^18^F-FDG reflects glucose hypermetabolism, a process common to both malignant cells and inflammatory tissues. Consequently, FDG imaging often fails to distinguish peritumoral inflammation from viable tumour tissue, potentially leading to an overestimation of tumour burden, particularly in the post-treatment setting where inflammatory responses are prevalent. Conversely, ^18^F-FLT serves as a specific marker of cell proliferation. Its retention is governed by Thymidine Kinase 1 (TK1), an enzyme upregulated strictly during the S-phase of the cell cycle. Although ^18^F-FLT generally exhibits lower absolute uptake due to the rate-limiting nature of TK1 phosphorylation [29, 30], its specificity for proliferating cells allows for a more accurate representation of tumour aggressiveness, devoid of inflammatory noise. This specificity is crucial for precise prognostication and treatment adaptation.

Previous Oesophageal cancer PET studies mainly centered on short - term efficacy and lymph node diagnosis, while ours focused on the prognostic values of PET/CT. Our multivariate analysis identified N3 stage as a significant negative prognostic factor for both PFS (*P* = 0.033) and OS (*P* = 0.021). The strong correlation between ^18^F-FLT lymph node parameters and survival suggests that ^18^F-FLT may offer superior accuracy in N-staging by identifying biologically aggressive nodal metastases that FDG might miss or conflate with reactive nodes. While ^18^F-FDG parameters showed no prognostic relevance, ^18^F-FLT primary tumour and lymph node parameters consistently predicted adverse outcomes. ROC curve analysis further corroborated the superiority of ^18^F-FLT. The AUCs for lymph node SUV _max_ and SUV _mean_ were significantly higher than those for primary tumour parameters. The detection of ^18^F-FLT-avid lymph nodes likely signifies active tumour proliferation and high invasiveness, which are precursors to distant metastasis. This biological underpinning explains why ^18^F-FLT lymph node parameters serve as more potent prognosticators than primary tumour metrics alone. Our findings contrast slightly with those of Chen et al. [31], who reported that in 34 ESCC patients, ^18^F-FLT was a superior predictor for 2-year PFS but not for OS. The discrepancy may be attributed to differences in treatment heterogeneity or follow-up duration between the cohorts. Nonetheless, our data reinforces the growing body of evidence [32–36] supporting ^18^F-FLT as a robust biomarker for long-term survival in various malignancies.

Using the Youden index, we determined optimal ^18^F-FLT PET/CT parameter cut-off values. These values effectively distinguished patients with favourable versus poor PFS and OS outcomes (all *P* < 0.05; Table 6), highlighting the potential of ^18^F-FLT PET/CT to stratify high-risk patients for personalized treatment. Clinically, such stratification is invaluable; patients with metabolic parameters exceeding these cut-offs may benefit from treatment intensification. Furthermore, the ability to detect metabolically active nodes aids precise radiotherapy targeting, enabling dose escalation to high-risk sub-volumes (dose painting) while sparing normal tissues—thereby optimizing the therapeutic ratio [24, 37].

Analysis of PFS subgroups revealed that ^18^F-FLT uptake parameters were significantly higher in patients with poor prognosis regardless of the examination timing relative to treatment initiation. Since nearly all patients were scanned within 2 weeks of treatment start, this suggests that ^18^F-FLT has the potential to identify poor responders at a very early stage, consistent with previous findings [32].

This study has several limitations. First, the sample size (*n* = 31) was relatively small, although the prospective design and exceptionally long follow-up partially mitigate this. Second, as a single-centre study, the external validity of the cut-off values requires confirmation in multi-centre trials. Third, the time interval between the ^18^F-FDG and ^18^F-FLT scans (as shown in Table 1) varied due to the additional time required for ^18^F-FLT preparation. This variation could theoretically introduce bias into the results. However, our prognostic group analysis demonstrated that this imaging timing did not significantly skew prognostic stratification. Additionally, the radiotherapy dose was not uniform (range 40–66 Gy), which is an inherent limitation of this real-world study and may have introduced some variability.

## Conclusion

Our findings suggest that ^18^F-FLT PET/CT may offer superior predictive value compared with ^18^F-FDG PET/CT for long-term prognosis in patients with locally advanced oesophageal squamous cell carcinoma. Specifically, parameters derived from ^18^F-FLT-positive lymph nodes, such as SUV _max_ and SUV _mean_, may serve as potential biomarkers for poor clinical outcomes. However, given the limitations of this study, these findings should be considered preliminary. Larger, multicenter studies are warranted to validate our results.

## Data Availability

The datasets generated and/or analysed during the current study are not publicly available due to patient privacy regulations but are available from the corresponding author on reasonable request.

## Abbreviations

^18^F-FDG: fluorine-18 fluorodeoxyglucose
^18^F -FLT: fluorine-18 fluorothymidine
AJCC: American Joint Committee on Cancer
AUC: Area Under the Curve
CCRT: Concurrent Chemoradiotherapy
CI: Confidence Interval
CT: Computed Tomography
EC: Oesophageal Cancer
ESCC: Oesophageal Squamous Cell Carcinoma
HR: Hazard Ratio
IMRT: Intensity-Modulated Radiation Therapy
LA-ESCC: Locally Advanced Oesophageal Squamous Cell Carcinoma
LN: Lymph Node
MTV: Metabolic Tumour Volume
NCRT: Neoadjuvant Chemoradiotherapy
OS: Overall Survival
PET: Positron Emission Tomography
PFS: Progression-Free Survival
ROC: Receiver Operating Characteristic
ROI: Region of Interest
SD: Standard Deviation
SUV: Standardized Uptake Value
SUV _max_: Maximum Standardized Uptake Value
SUV _mean_: Mean Standardized Uptake Value
SUV _min_: Minimum Standardized Uptake Value
TK1: Thymidine Kinase 1
TLG: Total Lesion Glycolysis
TLT: Total Lesion ^18^F-FLT Expression
TNM: Tumour, Node, Metastasis (staging system)
